# Hierarchical transcriptional control regulates *Plasmodium falciparum* sexual differentiation

**DOI:** 10.1186/s12864-019-6322-9

**Published:** 2019-12-03

**Authors:** Riëtte van Biljon, Roelof van Wyk, Heather J. Painter, Lindsey Orchard, Janette Reader, Jandeli Niemand, Manuel Llinás, Lyn-Marie Birkholtz

**Affiliations:** 1 Department of Biochemistry, Genetics and Microbiology, Institute for Sustainable Malaria Control, University of Pretoria, Private Bag x20, Hatfield, 0028 South Africa; 2 Present Address: Department of Biochemistry & Molecular Biology and the Huck Center for Malaria Research, Pennsylvania State University, University Park, PA 16802 USA; 3 Department of Biochemistry & Molecular Biology, the Huck Center for Malaria Research, University Park, PA 16802 USA; 4 Present Address: Division of Bacterial, Parasitic, and Allergenic Products, Center for Biologics Evaluation and Review, U.S. Food & Drug Administration, Silver Spring, MD 20993 USA; 5 Department of Chemistry, Pennsylvania State University, University Park, PA 16802 USA

**Keywords:** Malaria, *Plasmodium*, Gametocyte, Gametocytogenesis, Transcriptome, Gene expression regulation, Differentiation, Sexual development

## Abstract

**Background:**

Malaria pathogenesis relies on sexual gametocyte forms of the malaria parasite to be transmitted between the infected human and the mosquito host but the molecular mechanisms controlling gametocytogenesis remains poorly understood. Here we provide a high-resolution transcriptome of *Plasmodium falciparum* as it commits to and develops through gametocytogenesis.

**Results:**

The gametocyte-associated transcriptome is significantly different from that of the asexual parasites, with dynamic gene expression shifts characterizing early, intermediate and late-stage gametocyte development and results in differential timing for sex-specific transcripts. The transcriptional dynamics suggest strict transcriptional control during gametocytogenesis in *P. falciparum,* which we propose is mediated by putative regulators including epigenetic mechanisms (driving active repression of proliferation-associated processes) and a cascade-like expression of ApiAP2 transcription factors.

**Conclusions:**

The gametocyte transcriptome serves as the blueprint for sexual differentiation and will be a rich resource for future functional studies on this critical stage of *Plasmodium* development, as the intraerythrocytic transcriptome has been for our understanding of the asexual cycle.

## Background

Sustained malaria prevalence is ensured through continued human-to-mosquito transmission of *Plasmodium* parasites with *Plasmodium falciparum* being the causative agent of the most severe form of the disease in humans [1]. The complex life cycle of *P. falciparum* encompasses development in the liver and erythrocytes of its human host and transmission by the female anopheline mosquito. Two distinct developmental phases characterize intraerythrocytic development: rapid, cyclic asexual cell division manifesting in pathology, and the stochastic (< 10%) sexual differentiation into gametocytes [2, 3], which produces the non-replicative, mature, transmissible forms of the parasite. Whilst the intraerythrocytic developmental cycle (IDC) is relatively rapid (~ 48 h) and results in massive cell number expansion, sexual differentiation and development (gametocytogenesis) is a prolonged process (~ 10 days) in *P. falciparum* and is characterized by the development of the parasite through five morphologically distinct gametocyte stages (stages I-V) [4].

The processes of asexual replication and sexual differentiation in *Plasmodium* are associated with distinct patterns of gene expression that are tightly controlled through complex regulatory systems [5]. These patterns have been investigated to some extent for asexual replication where *P. falciparum* parasites use both transcriptional [6–8] and post-transcriptional processes [9, 10] to effect a cascade of coordinated, stage-specific gene expression [11, 12]. Despite the identification of some putative regulators of gene expression, including the Apicomplexan-specific ApiAP2 family of transcription factors [13–15] and epigenetic regulation of particular gene families [16, 17], the specific mechanisms controlling transcriptional activation in the parasite are incompletely understood, with recent data clearly showing mRNA dynamics are also influenced by additional post-transcriptional mechanisms [18, 19].

The mechanisms regulating commitment to gametocytogenesis have been somewhat clarified recently, with the discovery that host LysoPC restriction acts as an environmental factor driving gametocyte commitment [20]. The AP2-G transcription factor acts as a molecular master switch of sexual commitment [21–24] and results in the expression of genes that drive entry into gametocytogenesis [22–26]. The *ap2-g* gene is released from an epigenetically silenced state [27, 28] through the antagonism of heterochromatin protein 1 (HP1) epigenetic silencing of the *ap2-g* locus by the gametocyte development protein 1 (GDV1) [29]. Commitment to gametocytogenesis further requires stabilization of a subset of gametocyte-specific transcripts [18].

Despite these advances toward unravelling the mechanisms of commitment, the molecular functions governing subsequent gametocyte development and maturation remain poorly understood. Previously, deletion of certain ApiAP2 proteins has been shown to prevent progression of gametocyte development in the rodent parasite *P. berghei* [30] and *P. falciparum* [31]. Further, a subset of transcripts are translationally repressed by RNA binding proteins such as the Pumilio family protein (PUF2) during gametocytogenesis and ATP-dependent RNA helicase DDX6 (DOZI) and trailer hitch homolog (CITH) that repress female gametocyte transcripts needed to complete gametogenesis [32, 33]. However, systematic exploration of gene expression for *P. falciparum* gametocytogenesis has been limited to evaluation of the transcriptome [34–38] and proteome [35, 39–41] at specific developmental timepoints. This includes the bifurcation in committing asexual parasites to gametocytogenesis [18, 20, 25], and evaluation of mature gametocytes in preparation for transmission [35, 40, 41]. Current datasets that evaluate the complete gametocyte development process are sparse, with only one study that could detect 65% of the parasite’s transcriptome [36], which precludes dynamic evaluation of the transcriptomic profile associated with the extended gametocyte development process of *P. falciparum* parasites. Therefore, a time-resolved, high-resolution dataset capturing the transcriptome of each stage of gametocyte development would greatly enhance our ability to compare gene expression levels throughout the 10 days of gametocyte development and maturation.

Here we describe a comprehensive transcriptome analysis of *P. falciparum* parasites during all stages of sexual development at daily resolution. By measuring transcript abundance pre- and post-commitment, the transcriptional profile of gametocytes can be completely distinguished from that of asexual parasites. The data show marked shifts in transcript abundance associated with morphological stage transitions, indicating that gene expression occurs on a time scale consistent with developmental decisions underlying gametocyte development. We also show that post-commitment, the gametocyte transcriptome correlates to specific epigenetic marks and ApiAP2 transcription factors. The gametocyte transcriptome provides a quantitative baseline of gene expression throughout sexual development and constitutes a highly valuable resource that could be exploited to further understand the molecular mechanisms governing sexual differentiation and maturation of the malaria parasite.

## Results

### Transition between sexual and asexual stages of development defined by transcriptome

**Fig. 1:**
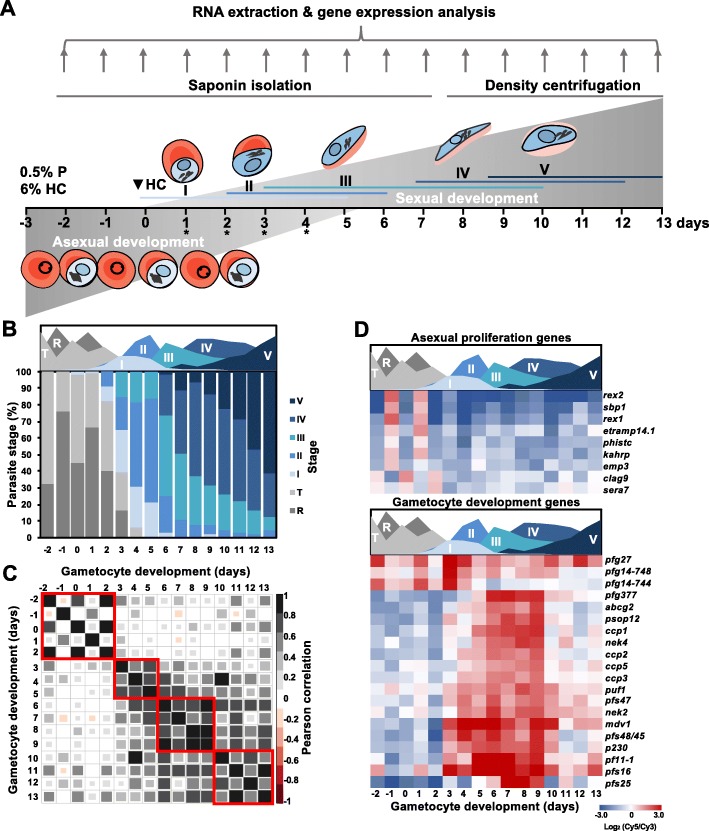
The developmental and associated transcriptomic profile of *P. falciparum* NF54 gametocytes from commitment to maturity. **a** Sampling and culturing strategy and stage distribution of parasites on each day of the time course. Colored lines indicate the presence of specific stages at different time points. Abbreviations indicate parasitemia (P) and hematocrit (HC) at induction, * indicate the addition of N-acetyl glucosamine (NAG) or 5% D-sorbitol. Parasite drawings were modified from freely available images (https://smart.servier.com/), under a Creative Commons Attribution 3.0 Unported Licence. **b** Morphological development was monitored from induction (day − 2) over 16 days of development using Giemsa-stained thin-smear microscopy. The stage distribution for each day was calculated by counting ≥100 parasites on each day of monitoring. Legend: I-V indicates different stages of gametocyte development, R = ring and T = trophozoite stage asexual parasites. **c** Pearson correlation coefficients of the total transcriptomes obtained for each day of development. Red boxes indicate localized phases of increased correlation. **d** Expression of “gold standard” asexual and gametocyte genes [43] are shown for the gametocyte time course in heatmaps. **a-d** Area plot designates the timing of appearance and abundance of specific stages throughout the time course

*P. falciparum* NF54-*pfs16*-GFP-Luc [42] parasites were induced to form gametocytes after 1.5 cycles of asexual development (3 days) and monitored for the next 13 days to capture gametocyte commitment and development to mature stage V gametocytes (Fig. 1a). Tight synchronization of the asexual parasites ensured coordinated gametocyte development, and gametocytes (stage I) were observed in culture from day 0 onwards (Fig. 1b). Morphological evaluation showed a shift from a predominantly asexual parasite population to > 60% gametocytes by day 3 of gametocytogenesis following the removal of asexual stages (Fig. 1b).

We measured mRNA abundance genome-wide using DNA microarrays that included 5792 annotated transcripts, each represented by > 2.5 probes including ncRNA and tRNAs that could produce unique probes [44]. Using two color arrays in which the Cy5 channel pooled total RNA from timepoints comprised of both asexual and sexual stages, we were able to easily normalize each gene at every timepoint to distinguish the timing of peak abundance throughout the entire time course and across developmental stages. In each sample, expression values were captured for 96–99% of the 5443 genes on the array (*P* < 0.01, full dataset provided in Additional File 1), a 1.5-fold improvement in coverage compared to the 65% of the transcriptome (3410 genes) captured in the previously reported Young et al. dataset [36]. Overall, the transcriptome of gametocytes is distinct from asexual parasites, as is evidenced by a clear shift in Pearson correlation between the transcriptomes of asexual parasites (day − 2 to 2) and gametocytes (day 3 onward) (Fig. 1c). Populations containing predominantly asexual parasites (days − 2 to 2) were highly correlated across the first two 48 h cycles (r^2^ = 0.54–0.86, data provided in Additional File 2) and were characterized by periodic gene expression changes between the asexual ring and trophozoite stages (Fig. 1c). From Day 3 onward, the transcriptional profiles diverged indicating a switch from asexual to sexual development, evidenced by a loss of the 48 h correlation pattern (Fig. 1c). During subsequent days of gametocytogenesis, daily peak correlations were associated with developmental progression through stage I-II (days 3–5, r^2^ = 0.56–0.73), stage III-IV (days 6–9, r^2^ = 0.51–0.92), and mature stage V gametocytes (days 10–13, r^2^ = 0.50–0.84) (Fig. 1c, data provided in Additional File 2), which corresponded to morphological transitions observed via Giemsa-stained thin-blood smears throughout the time course.

Conversion from asexual to sexual development was also clearly detectable in the expression profiles of individual genes required during asexual development (e.g. *kahrp (pf3d7_0202000)*) while sexual genes were only expressed during gametocyte development from Day 3 [43] (Fig. 1d). The genes restricted to expression during sexual development include downstream targets of PfAP2-G [23] and markers associated with mature gametocyte sex-specificity (Fig. 1d) [35] and 24 novel gametocyte-associated transcripts (data provided in Additional File 2). Among these transcripts were a putative ncRNA, three rRNAs and two tRNAs, suggesting that the expression of non-coding RNAs may not only play a role during gametocyte commitment [18] but also in gametocyte development and maturation in *P. falciparum.* Together, these data comprise a high-resolution *P. falciparum* blood stage developmental transcriptome that allows for the temporal evaluation of transcriptional abundance patterns associated with gametocyte commitment, development and maturation.

### The gametocyte-specific transcriptional program reflects the molecular landscape of gametocyte development

**Fig. 2:**
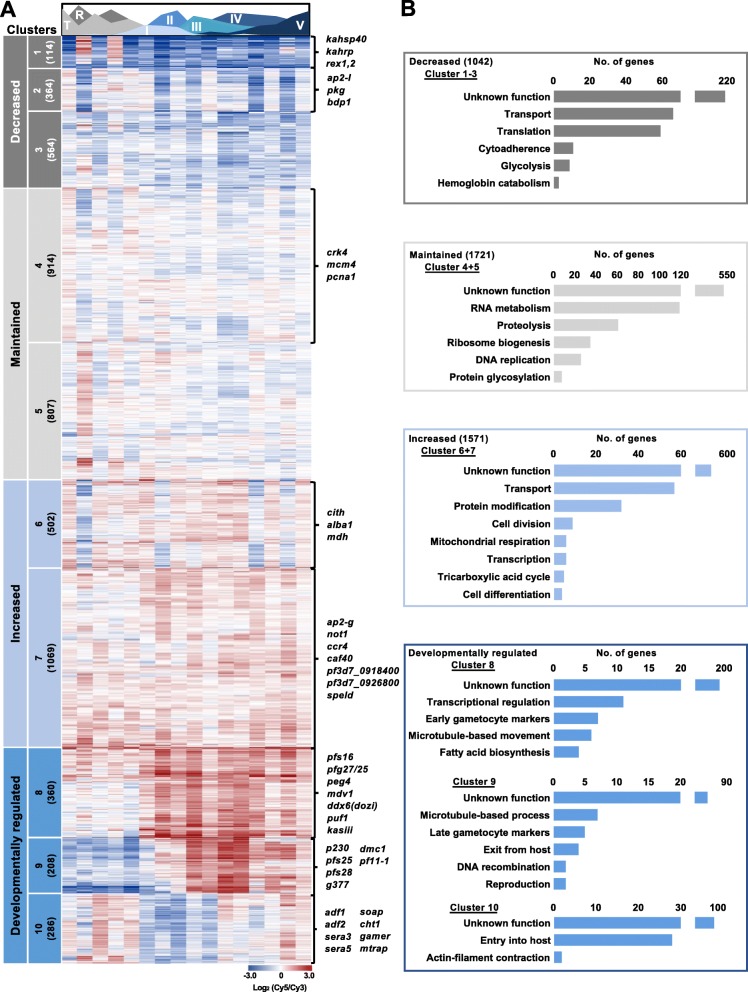
Distinct clusters of expression link to biological development of the *P. falciparum* gametocyte. Clusters of genes expressed during gametocyte development following K10 clustering of the total transcriptome. **a** The 10 clusters were grouped into phases of decreased, maintained, increased or developmentally regulated transcript abundance with number of transcripts per cluster indicated in brackets and genes of interest from specific clusters highlighted next to heatmaps. Area plot designates the timing of appearance and abundance of specific stages throughout the time course. **b** Biological processes of interest were selected from GO enrichment (Additional File 1) of each of the clusters (*P <* 0.05) with the number of genes related to these functions shown for the groups of clusters in bar graphs with generic descriptions of the gene sets used to describe their function

To associate temporal gene expression to gametocyte commitment and stage transitions throughout development, the full 16-day transcriptome dataset was K-means clustered revealing 2763 transcripts with overall decreased abundance (clusters 1–5) and 2425 with increased abundance during gametocytogenesis (clusters 6–10, Fig. 2a). Therefore, gametocytogenesis relies on a more specialized program of gene expression compared to asexual development, with only 45% of transcripts showing increased abundance during gametocyte development (Fig. 2a) compared to the 80–95% of transcripts increased during specific phases of asexual development [11, 19, 45]. Interestingly, individual clusters showed specific patterns of gene expression throughout gametocyte development (Fig. 2a), with transcript abundance during gametocytogenesis either decreased following asexual development (clusters 1–3, 1042 transcripts); maintained (clusters 4–5, 1721 transcripts) or increased (cluster 6–7, 1571 transcripts). Three clusters (clusters 8–10) show transcripts with specific peaks in expression during development, indicative of developmental gene regulation.

Cluster 1 is predominantly comprised of critical asexual stage transcripts which showed a decline in abundance during gametocytogenesis, with up to 5 log_2_ fold (log2FC (Day3/Day1)) decreases in the expression of these transcripts between the ring and early gametocyte stages (Fig. 2a). These transcripts include Maurer’s cleft proteins e.g. *rex1 (pf3d7_0935900)* and *rex2 (pf3d7_0936000)* as well as knob associated proteins that form part of the cytoadherence complex *(kahrp (pf3d7_0202000), kahsp40 (pf3d7_0201800))*, supporting earlier observations that the gametocytes mediate sequestration via different mechanisms than asexual parasites [46]*.* Many of these cytoadherence associated transcripts are associated with heterochromatin protein 1 (HP1) occupancy during gametocyte development [47], and other HP1 [47, 48] and H3K9me3 [17] repressed genes are also significantly enriched in cluster 1 (*P <* 0.0001, Fisher’s exact test, genes listed in Additional File 3). This suggests asexual development-specific genes are actively repressed by epigenetic regulation throughout gametocyte development. Clusters 1–3 also contain transcripts involved in metabolic processes that are not critical to gametocyte development including genes encoding for enzymes of heme metabolism and glycolysis (Fig. 2b, cluster 3, Additional File 1) as well as regulators of egress (*pkg (pf3d7_1436600)*) and invasion (*bdp1 (pf3d7_1033700)* and *ap2-i* (*pf3d7_1007700)*), all processes that are not necessary for gametocyte maturation (Fig. 2a, cluster 2). Beyond these examples, clusters 1–3 also contain 214 unannotated genes that could be specifically required for asexual development only (Fig. 2b).

Some transcripts show low abundance throughout gametocytogenesis (Fig. 2a, clusters 4 and 5, average expression < 0.1 log_2_(Cy5/Cy3), with amplitude change < 0.5 log_2_(Cy5/Cy3)). These clusters include regulators of proliferation (e.g. origin of replication complex protein *mcm4 (pf3d7_1317100),* proliferating cell antigen 1 (*pf3d7_1361900)* and cyclin dependent kinase *crk4 (pf3d7_0317200*)). By comparison, clusters with transcripts maintained at increased levels throughout commitment and development (Fig. 2a, clusters 6 and 7, average log_2_(Cy5/Cy3) > 0.31) included expected gene sets involved in the constitutive processes of macromolecular metabolism (e.g. DNA replication, protein modification and RNA metabolism Fig. 2b, Additional File 1) [36, 38]. Interestingly, cluster 6 (and cluster 2) showed a high degree of cyclic oscillation in transcript abundance (Fig. 2a). Many of these transcripts relate to transport, general cellular metabolism and homeostasis, functions in which fluctuation would not be unexpected (Fig. 2b, Additional File 1). Importantly, cluster 7 also contained transcripts classified by gene ontology as involved in cellular differentiation *(caf40 (pf3d7_0507600)*, *pf3d7_0918400, pf3d7_0926800* and *speld (pf3d7_1137800))* (GO:0030154, *P =* 0.026, Fig. 2b, S1 Table).

A significant proportion (15%) of the transcriptome is associated with peak expression during specific stage-transitions in gametocyte development (Fig. 2a, clusters 8–10), reminiscent of the phased expression typical of the asexual transcriptome [11, 12]. Transcripts involved in early-stage development increased from stage I-II in cluster 8 in a transcriptional profile often associated with targets of AP2-G [22, 23, 25]. Transcripts in cluster 9 increased in abundance in the intermediate phase of development (stage III-IV) before the expression of transcripts required for development in mosquitoes in cluster 10 (stage V, *gamer* (*pf3d7_0805200*), *mtrap* (*pf3d7_1028700*), *cht1* (*pf3d7_1252200*), Fig. 2a &b). The transcripts in clusters 8–10 are thus markers of biological transitions during gametocyte development. Clusters 6 & 8 are enriched for genes that contribute to the metabolic shift to mitochondrial metabolism (e.g. malate dehydrogenase *(mdh, pf3d7_0618500))* and fatty acid biosynthesis (e.g. β-ketoacyl-ACP synthase III (*kasIII, pf3d7_0618500))* [49, 50] in gametocytes, followed by the emergence of processes related to cytoskeletal formation (clusters 8 & 9, Fig. 2a &B, Additional File 1: Table S1) that lead to the construction of a rigid subpellicular microtubule array during the sequestering stages (stages I-IV) of gametocytes [51]. The microtubule array results in the characteristic crescent shape of the intermediate stages before the complex depolymerizes in stage V that is accompanied by the increased transcript abundance of actin depolymerization factors 1 and 2 (Fig. 2b, cluster 10, *pf3d7_0503400, pf3d7_1361400*) to allow for a more deformable erythrocyte that can re-enter circulation [51]. This cluster also includes the genes encoding the serine repeat antigens (*sera*) 3 and 5 *(pf3d7_0207800, pf3d7_0207600)* that play a role in egress in asexual parasites [52, 53], implying that they may retain this role during gametocyte egress from the erythrocyte in the mosquito midgut. The striking temporal patterns of transcript abundance in clusters 8–10 suggests strict transcriptional regulation of these genes to ensure the timing of gametocyte sequestration, circulation and egress. Interestingly, these patterns are exhibited by parasites that need not fulfil any of these functions when grown in vitro in the absence of host-interactions, suggesting that transcription of these genes is hard-wired*.*

### Different gene sets enable sexual commitment and development

**Fig. 3:**
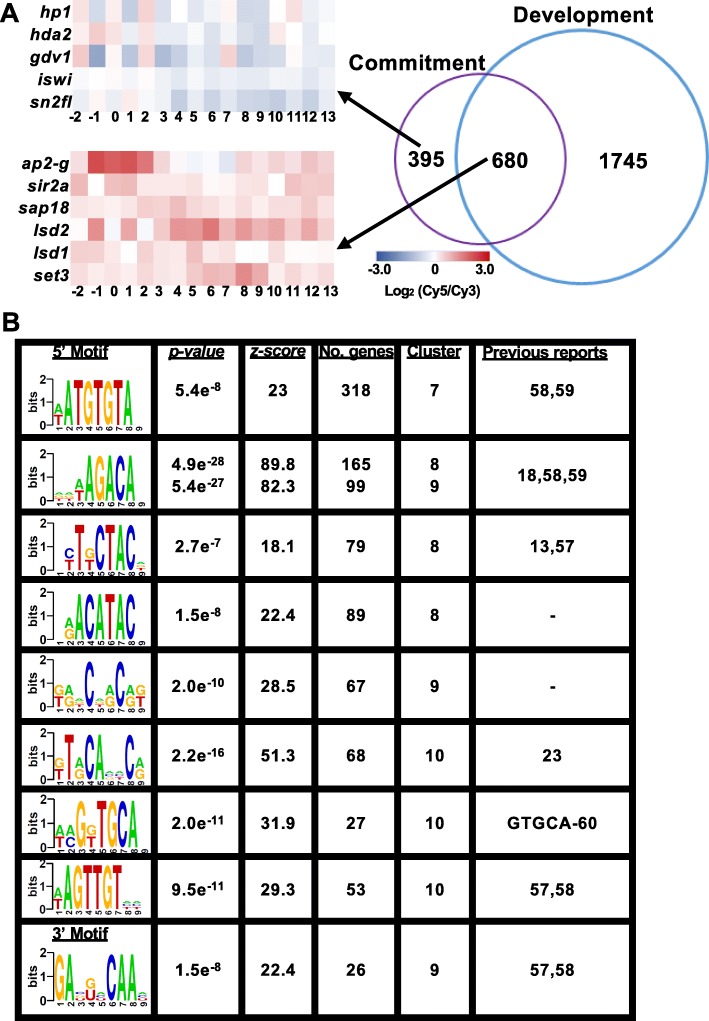
Commitment and development are distinctly regulated processes. **a** The genes increased in expression during commitment [18, 20, 25] were compared to transcripts increased in abundance during gametocytogenesis (Clusters 6–10, 2425 transcripts) with overlapping genes of interest: *ap2-g (pf3d7_1222600), sap18 (pf3d7_0711400), sir2a (pf3d7_1328800), lsd2 (pf3d7_ 0801900), lsd1 (pf3d7_ 1,211,600), set3 (pf3d7_ 0827800)* and genes only increased during commitment *hp1 (pf3d7_1220900), hda2 (pf3d7_1008000), gdv1 (pf3d7_0935400)*, *iswi (pf3d7_0624600), sn2fl (pf3d7_1104200)* highlighted in heatmaps. **b** The increased and developmentally regulated gene clusters also contained significantly enriched regulatory 5′ and 3′ UTR motifs identified using the FIRE algorithm [54]

The time-resolved gametocyte transcriptome also allows interrogation of the expression of genes involved in sexual commitment throughout gametocyte development [18, 20, 25] (Fig. 3). In total, previous reports produced a set of 1075 unique genes proposed to function as an “on switch” that characterizes gametocyte commitment [18, 20, 25]. Of these, 680 genes (63%) also have increased transcript abundance during gametocyte development (Fig. 3). These increased transcripts include those encoding epigenetic regulators involved in cell cycle control such as SIR2A (PF3D7_1328800) and SAP18 (PF3D7_0711400) that contribute to decreased DNA synthesis and a block in proliferation [55, 56] necessary for the parasite to differentiate (Fig. 3a) as well as other epigenetic regulators LSD1,2, SET3 (PF3D7_0801900, PF3D7_1,211,600, PF3D7_0827800). These epigenetic modifiers and readers do not have direct roles postulated for commitment, but could contribute to the global change in abundance of specific histone marks as the parasite differentiates [57]. The remaining 395 transcripts are not increased in abundance during gametocyte development, suggesting that these transcripts are short lived and possibly only essential during gametocyte commitment. These short lived transcripts include *gdv1,* whose protein product prevents epigenetic repression of *ap2-g* during commitment [29], *iswi* and *sn2fl*, which encode chromatin remodelling proteins (Fig. 3a)*,* that are expressed in sexually committed cells downstream of *ap2-g* [25] and *hp1* and *hda2* that antagonise *ap2-g* expression [27].

From our data, we also identified specific 5′ *cis*-regulatory motifs that are enriched upstream of genes involved in gametocytogenesis (Fig. 3b). The first motif, (ATGTGTA) was highly represented in cluster 7 in genes that express ubiquitously throughout both sexual and asexual development. This motif has been correlated with genes involved in DNA replication [54] and the significance of its enrichment in genes associated with differentiation is unclear. The second motif, (AGACA) that is enriched upstream of the genes in the developmentally regulated clusters 8 and 9 has been associated with sexual commitment and development in previous datasets [18, 58] although no *trans-*acting factors have been identified for either of these motifs [15, 59] (Fig. 3b). Additionally, a second well-conserved motif was enriched in cluster 8, (ACATAC) that has not been reported before and possibly represents a new avenue for investigation of *cis-*regulatory elements in genes contributing to parasite differentiation. In addition, genes in cluster 10 were enriched for 3 motifs, of which the first (GT[A/G]CA) closely matches the composite motif observed in genes bound by both the AP2-I and AP2-G transcription factors [23] and the second motif (GGTGCA) closely resembles the transcription factor binding site of AP2-I alone [60]. Cluster 9 was the only cluster of genes with an enriched motif in their 3′ UTR, coinciding with 63% of this cluster being translationally repressed in the gametocyte stage [32, 35].

### Transcriptional patterns characterize distinct transitions in gametocyte development

**Fig. 4:**
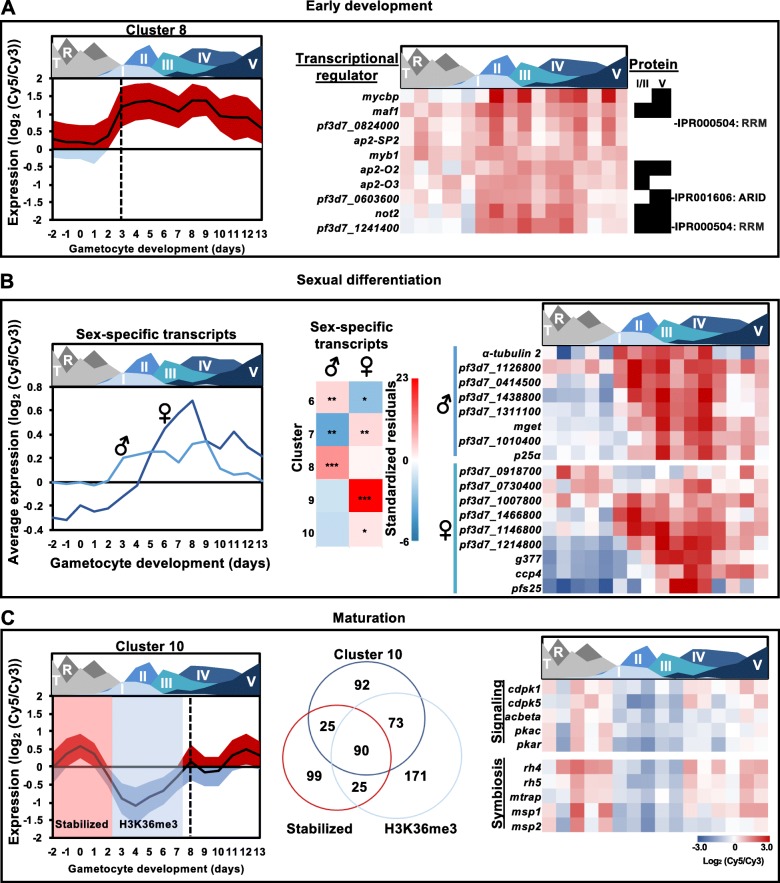
Stage-specific increases in gene expression contribute to the extended differentiation of *P. falciparum* gametocytes. **a** During stage I-III of development genes in cluster 8 sharply increased in expression (indicated with dotted line) with the abundance of these transcripts indicated by ribbon plot with mean ± SD. GO enrichment of genes involved in regulation of transcription (GO:0010468, 11 transcripts, *P =* 0.029) is present in this cluster, with presence of protein for these genes in stage I/II and V indicated in black [35, 39–41] and the corresponding Interpro domains (https://www.ebi.ac.uk/interpro/) of proteins with unknown function indicated on the right. **b** The timing of sexually dimorphic transcript profiles [35] are shown in line graphs while the association of male-and female-enriched transcripts with specific clusters [6–10] are shown as standardized residuals and significance of these associations indicated (*P <* 0.05*,0.001**,0.0001***, Fisher’s exact test). Genes of interest for each sex are highlighted in heatmaps next to male and female symbols. **c** The genes expressed during maturation (cluster 10) showed a significant association (*P <* 0.0001, two-tailed Fisher’s exact test) with genes stabilized post-transcriptionally during commitment [18] and H3K36me3-associated genes in asexual development [16, 61] before a sharp increase at stage IV-V of development (dashed line). Blocks indicate the timing of stabilization of the transcripts [18] or abundance of the H3K36me3 mark [57] and the overlap between the 3 datasets are indicated in the Venn diagram. Genes of interest within the three functional datasets are highlighted in heatmap. **a-c** Area plot designates the timing of appearance and abundance of specific stages throughout the time course

Apart from commitment to sexual development, the parasite also undergoes distinct developmental and transcriptional transitions during gametocyte development. The initial transition occurring in stage I gametocytes and regulating immature gametocyte development is characterised by increased transcript abundance in cluster 8 (Fig. 4a), which showed a significant enrichment for genes involved in regulation of transcription (GO:0010468, 11 transcripts, *P =* 0.029) including the specific ApiAP2 transcription factors *pf3d7_0404100, pf3d7_0516800, pf3d7_1429200* and the *myb1* transcription factor (*pf3d7_1315800)* (Fig. 4a). Other genes with potential regulatory functions include a possible novel transcription factor, *pf3d7_0603600*, which contains an AT-rich interaction domain (IPR001606: ARID) and an uncharacterized RNA binding protein *(pf3d7_1241400)*. Proteins expressed by these two genes have been detected previously during gametocyte development (Fig. 4a) [34, 35, 40, 41]. These proteins, along with the C-Myc binding protein MYCBP (PF3D7_0715100), are of interest for further study to determine their role in controlling gene expression during gametocyte development.

A second outcome of the initial transition into gametocytogenesis is the determination of sex differentiation in *P. falciparum* parasites, which is proposed to be an PfAP2-G independent process that occurs at the very onset of commitment [18, 35, 40, 41, 62]. However, sexually dimorphic gametocytes are only morphologically detectable by microscopy from stage III onwards [63]. Our data indicate that the male-enriched transcripts from Lasonder et al. 2016 [35] show increased abundance earlier in development (stage I-II; 27% of cluster 8, *P <* 0.0001, two-tailed Fisher’s exact test, Fig. 4b, Additional File 3) compared to female transcripts. These 98 male-enriched transcript abundance may be good biomarkers of early male differentiation as an alternative to α-tubulin II, which is expressed promiscuously in early gametocyte populations [64].

Female-enriched transcripts [35] peak in abundance only after sexual dimorphism is clearly discernible, from stage II-III onwards (Fig. 4b) and are significantly overrepresented in the intermediate development cluster 9 (Fig. 4b, Additional File 3, 76% of the cluster, *P <* 0.0001, two-tailed Fisher’s exact test). Overall, this trend held true for the 158 female-enriched transcripts in cluster 9, including those encoding canonical female markers, e.g. osmiophilic body protein *g377* (*pf3d7_1250100)* [65, 66], late-stage antigen *pfs25* (*pf3d7_1031000)* [35, 66] and *ccp1–3 (pf3d7_1475500, pf3d7_1455800, pf3d7_1407000)* [35, 66] and *ccp4 (pf3d7_0903800)* that was recently used to reliably type male and female gametocytes in late-stage gametocytes [67]. We also detect a small subset of female-enriched transcripts (*pf3d7_0918700, imp2 (pf3d7_0730400), pf3d7_1007800, pf3d7_1466800, pf3d7_1146800, obc13 (pf3d7_1214800))* that are expressed earlier in gametocyte development (Fig. 4b) and could potentially be important for female development before morphological differences are apparent.

The second transcriptional transition we observed coincides with the onset of gametocyte maturation from stage IV to V (Fig. 4c). These transcripts show increased abundance in sexually committed asexual parasites as well as mature stage V gametocytes but have diminished abundance during the early and intermediate stages of gametocytogenesis (cluster 10, Fig. 4c). This cluster was significantly enriched for transcripts stabilized during commitment (47% of transcripts, *P* < 0.0001, two-tailed Fisher’s exact test) [18], as well as genes marked with H3K36me3 in asexual parasites (49% *P* < 0.0001, Fisher’s exact test) [16]. Interestingly, the epigenetic H3K36me3 mark is abundant during the intermediate stages of gametocyte development [57] and genes overlapping in the three datasets encode transcripts associated with the intracellular signalling machinery of the parasite (*cdpk1 (pf3d7_0217500), cdpk5 (pf3d7_1337800)* and adenylyl cyclase beta *(pf3d7_ 0802600,* [68])*,* along with cAMP-dependent protein kinase A catalytic and regulatory subunits (*pkac (pf3d7_0934800), pkar (pf3d7_1223100)* (Fig. 4c). Of these, CDPK1 has been confirmed to function in de-repressing female gametocyte transcripts during parasite development in mosquitoes [69]. Several of the genes in cluster 10 also have roles in invasion including the merozoite proteins *msp1, pf3d7_0930300, msp2, pf3d7_0206800, rh4, pf3d7_0424200,* and *rh5, pf3d7_0424100*, suggesting that invasion genes need to again be expressed for transition to gametogenesis in the mosquito. Overall, the gametocyte transcriptome reveals three major stages in gametocyte development (differentiation (Fig. 4a), intermediate development (Fig. 4b), maturation (Fig. 4c)) that promote gametocyte maturation of *P. falciparum* parasites.

### ApiAP2 transcription factors are expressed at specific intervals during gametocytogenesis

**Fig. 5:**
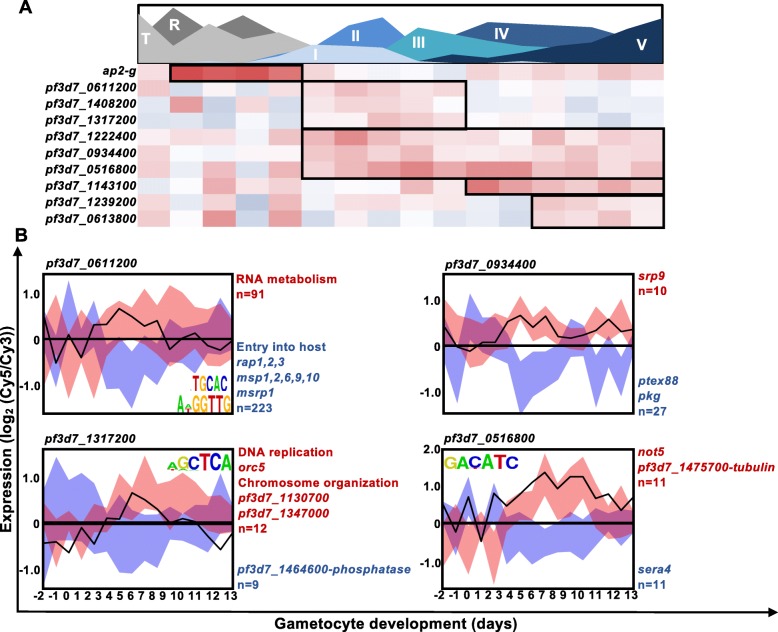
ApiAP2 transcription factors act as regulatory elements during gametocytogenesis. **a** ApiAP2 transcription factors increased in transcript abundance during gametocytogenesis were evaluated for their expression throughout gametocyte development with blocks indicating periods of increased abundance. Area plot designates the timing of appearance and abundance of specific stages throughout the time course. **b** The transcription factors were also probed for regulatory activity using coexpression analysis by GRENITS. Transcription factors with known binding sites [13], were probed against genes containing the transcription factor binding sites indicated or the total transcriptome if their binding site was unknown. The targets of each transcription factor are shown by shaded ribbons, with correlated transcripts indicated in red and anticorrelated transcripts indicated in blue. Newly identified motifs were associated with genes coexpressed with *pf3d7_0611200* using the FIRE algorithm [54] and are indicated on the graph. Generic functional terms describing enriched gene ontology terms or individual gene products are indicated in red (increased transcripts) or blue (decreased transcripts)

To investigate the possible contribution of factors associated with transcriptional regulation to the observed stage-progressions during gametocytogenesis, we interrogated the expression of the genes encoding the ApiAP2 transcription factor family (Fig. 5). Of the 27 family members, 15 genes encoding ApiAP2 transcription factors increased in transcript abundance during gametocyte development. Transcript abundance for *pf3d7_0404100, pf3d7_1350900, pf3d7_1449500, pf3d7_0802100, pf3d7_1429200* increased consistently throughout the time course (Additional file 4: Fig. S1). However, most ApiAP2-encoding transcripts increased in abundance at discrete intervals (Fig. 5a) throughout gametocytogenesis. As expected, *ap2-g (pf3d7_1222600)* transcript abundance increased before the appearance of gametocytes (days − 1 to 2). The target genes bound by AP2-G [23], peaked in transcript abundance directly following AP2-G peak abundance as expected, coinciding with stage I of gametocyte development (Additional Figs. 2 & 3). Thereafter, three transcription factors *pf3d7_1408200, pf3d7_1317200* and *pf3d7_0611200* were increased during stage I to III of development (days 2–6). In the rodent-infectious malaria parasites *P. berghei* and *P. yoelii*, orthologs of the first two genes have been associated with gametocyte development through knockout studies [21, 30, 70] while little is known of *pf3d7_0611200*. Three ApiAP2-encoding transcripts for *pf3d7_0516800, pf3d7_1222400, pf3d7_0934400* were increased in abundance from stage I to V of development (Fig. 5a), following a pattern similar to the increased abundance of cluster 8 (Fig. 4a). During the later stages, *pf3d7_1143100, pf3d7_1239200* and *pf3d7_0613800* were increased in abundance. Expression of *pf3d7_1143100* is translationally repressed in *P. berghei* gametocytes [32], indicating that these transcription factors may not contribute to gene expression in *P. falciparum* gametocytes, but may instead have functional significance in subsequent development in the mosquito.

To associate functional regulation of gene sets due to the cascade-like increased abundance of the transcripts encoding the ApiAP2 transcription factors, the gametocyte transcriptome was analysed using Gene Regulatory Network Inference Using Time Series (GRENITS [71]) (Additional File 1) with the strongest predicted regulators shown in Fig. 5b. From this analysis, *pf3d7_0611200,* which increased in abundance directly following *ap2-g,* coexpressed with 314 genes (probability linkage > 0.6), 223 of which were anti-correlated for expression and functionally enriched for genes involved in host invasion (GO:0044409: entry into host, *P =* 2.97e^− 12^; Fig. 5b). Interestingly, 116 of the genes co-expressed with this transcription factor were enriched for the TGCAC motif (*P* = 5.5e^− 13^), of which 100 were negatively co-expressed, indicating a repressive role for this transcription factor, either alongside or instead of the *P. falciparum* ortholog of *pbap2-g2* [21], *pf3d7_1408200.* This motif bears a striking resemblance to the motif bound by the 3rd AP2-domain of AP2-I, GTGCAC [13], suggesting this transcription factor could act as repressor of the invasion genes that AP2-I activates in asexual development. A secondary enriched domain was present in 43 of the co-expressed genes (GGTTG) and both of these binding motifs warrant further study into their functional relevance. The second *apiap2* transcript increased in abundance, is *pf3d7_1317200,* the *P. falciparum* ortholog of *pbap2-g3,* coexpressed with 21 genes involved in cell cycle processes, including DNA replication (GO:0044786, *P =* 0.0061) and chromosome organization (GO:0051276 *P =* 0.0046). Unlike its ortholog in *P. berghei* [70]*,* no enrichment for female specific proteins or transcripts were observed in the co-expressed genes and further phenotypic information is needed to describe this ApiAP2’s activity in *P. falciparum.* The two final ApiAP2 transcription factors are increased between stage I-V of development, with the first, *pf3d7_0934400,* showing mostly negative co-expression with its target genes (27/37 transcripts, including *pkg* and *ptex88 (pf3d7_1105600)*), suggesting this ApiAP2 transcription factor might also act as repressor. Secondly, the transcript of *ap2-o2* is increased in abundance throughout development but peaks at stage IV (day 8–9) of development and was predicted to regulate 22 target genes. Taken together, this data supports the involvement of successive expression of ApiAP2 transcription factors in a regulatory cascade during gametocyte development, as has been proposed for *P. berghei* gametocytes [21] and shows that this subsequent expression co-occurs with stage transition during *P. falciparum* gametocytogenesis.

## Discussion

We describe a high-resolution gametocyte transcriptome of malaria parasite differentiation from the asexual form through sexual commitment and all stages of development to mature stage V gametocytes. This dataset is currently the most comprehensive and reliable description of the changes in the transcriptome during the complete process of *P. falciparum* gametocytogenesis and presents a unique resource to the malaria research community. The dataset has and almost complete coverage of the transcriptome (~ 5400 genes) for the entirety of gametocyte development compared to only 100 s of transcripts currently detected with scRNA-seq experiments on a single stage of gametocyte development/ commitment [25, 66, 72]. This data further allowed in-depth analysis of the transcriptome and revealed novel findings, which was not detectable in previous lower resolution [36] or stage-focused datasets [37].

**Fig. 6:**
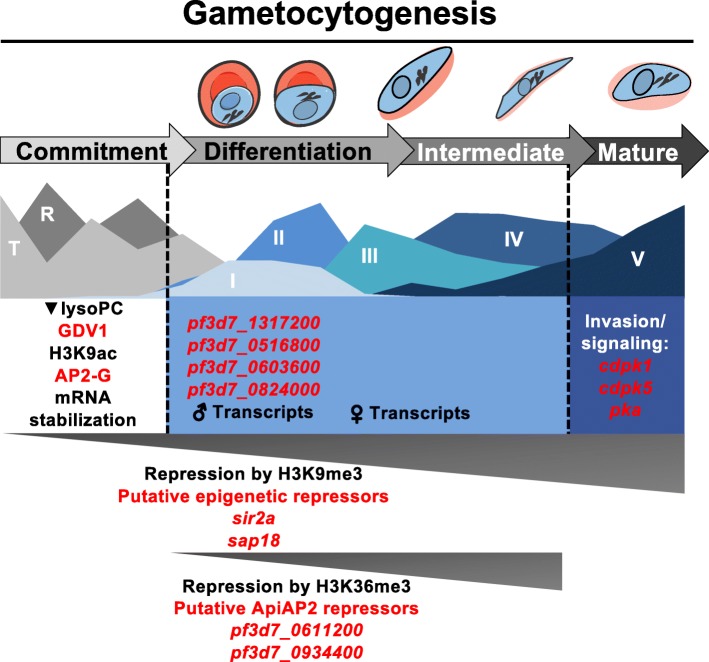
Molecular model of regulatory modules that shape cellular differentiation during gametocytogenesis. Specific regulatory events are mapped out over the extended gametocyte development of *P. falciparum* parasites. Molecular regulators are highlighted in red while specific events or epigenetic marks are shown in black. Colored blocks indicate the span of specific phases of transcript abundance, with dotted lines indicating transition points in gametocyte development and grey triangles indicate the timing of repressive mechanisms in gametocyte development. Parasite drawings were modified from freely available images (https://smart.servier.com/), under a Creative Commons Attribution 3.0 Unported Licence

We find that gametocytogenesis in *P. falciparum* is a well-controlled process involving successive activation of regulatory processes that mediate development during stage-transition, ultimately resulting in a parasite poised for transmission. These observations emphasize that stage-specific gene expression is an essential feature of regulation of gene expression in *Plasmodium* spp. and is particularly true for the extended and morphologically diverse gametocyte development of *P. falciparum* parasites. The dynamic evaluation of the transcriptome allows for the construction of a more complete molecular roadmap for gametocyte development (Fig. 6).

We propose that multiple transition points are passed during sexual differentiation and that mechanisms independent of initial sexual commitment during asexual proliferation are needed to ultimately result in completion of gametocyte development. First, committed parasites pass an initial transition point, whereby processes are initiated to drive early gametocyte development. This includes repression of genes typically associated with proliferation through epigenetic mechanisms (H3K9me3, HP1 occupancy), post-transcriptional regulation [18] and the activity of transcription factors that repress asexual-specific transcription (Fig. 6). This transition point is also characterized by the increased transcript abundance of genes required during early and intermediate gametocyte development. A portion of these transcripts are expressed specifically in either sex, with an apparent delay between the peak abundance of male-specific and female-specific transcripts (Fig. 6). It is possible that tracing the transcriptional dynamics within each sex separately would result in higher resolution data for this observation and resolve whether this is a true delay or underplayed by more complicated transcriptional dynamics that impact the RNA biology of the disparate sexes. As the gametocyte then reaches the critical transition for its pathology, the point of gametocyte maturation, a different set of genes increase in abundance. These genes are active in important processes specific to maturation into stage V gametocytes, including the switch from sequestration in the bone marrow to re-entering circulation and readying for transmission to the mosquito [73] by involving the parasite’s intracellular signalling machinery.

A particularly interesting observation is the decreased abundance of important regulators of commitment, *ap2-g* and *gdv1,* as the parasite enters the early gametocyte stages (Fig. 6). It is possible that the limited activity of these regulators might be essential for gametocytogenesis to occur normally, to allow the distinct patterns of gene expression we see here. It would be of interest to test what the effect of overexpression of one or both factors would be on the extended gametocyte development of *P. falciparum* parasites. We also add to data on the transcriptional regulation in *P. falciparum* by the ApiAP2 transcription factor family downstream of AP2-G, affirming the presence of a transcription factor cascade enabling passage through gametocytogenesis as postulated for *P. berghei* [21]*.* The involvement of *ap2-g2* and *pf3d7_0611200* in repressing transcription of asexual genes during gametocyte development (Fig. 6) is also of particular interest for investigation, bringing into question if one or both factors fulfil this role in *P. falciparum* gametocytes. However, the possibility of novel regulators of transcription in early gametocyte development cannot be overlooked, with RNA binding proteins and the possible ARID transcription factor (Fig. 6) good candidates for functional characterization.

## Conclusions

The high-resolution transcriptome profile of *P. falciparum* gametocytes offers a complete molecular landscape of parasite differentiation. We identify putative regulators of mRNA dynamics facilitating a well-timed transcriptional program that prepares the parasite for transmission. The profile provides molecular identity to differences and similarities in asexual and sexual development that can be exploitable for pharmaceutical intervention. Finally, the stage-specific events that complicate transmission-blocking drug discovery are highlighted, 1) the immediate divergence of the gametocyte’s molecular profile from asexual development, 2) the later sexual dimorphism in intermediate stage development and 3) the apparent transcriptional divergence between immature and mature gametocytes. The gametocyte transcriptome further provides a valuable resource for further interrogation of the function of gene products and regulatory mechanisms important for gametocytogenesis in *P. falciparum.*

## Methods

### Parasite culturing and sampling

In vitro cultivation of intraerythrocytic *P. falciparum* parasites and volunteer blood donation for human erythrocytes holds ethics approval from the University of Pretoria University of Pretoria Faculty of Natural and Agricultural Sciences Ethics Committee (EC120821–077). Human erythrocytes were obtained from volunteer donors after written informed consent was provided. Asexual *P. falciparum* NF54 parasite cultures (NF54-*pfs16*-GFP-Luc, a kind gift from David Fidock, Columbia University, USA [42]) were maintained at 5–8% parasitemia 37 °C in human erythrocytes at 5% hematocrit in RPMI 1640 medium supplemented with 25 mM HEPES, 0.2% D-glucose, 200 μM hypoxanthine, 0.2% sodium bicarbonate, 24 μg/ml gentamicin with 0.5% AlbuMAX® II and incubated under hypoxic conditions (90% N_2_, 5% O_2_, and 5% CO_2_) [74]. Synchronous asexual cultures (> 95% synchronized 5–10 hpi ring-stage parasites) were obtained by three consecutive cycles of treatment with 5% D-sorbitol, each 6–8 h apart.

Gametocytogenesis was induced by employing a strategy of concurrent nutrient starvation and a decrease of hematocrit [74]. Ring-stage parasite cultures were adjusted to a 0.5% parasitemia, 6% hematocrit in RPMI 1640 medium prepared as for growth of asexual parasites without additional glucose supplementation (day − 3) and maintained under the same hypoxic conditions at 37 °C without shaking. After 72 h, the hematocrit was adjusted to 3% (day 0). After a further 24 h, induction medium was replaced with medium containing 0.2% (w/v) D-glucose as the asexual parasites were removed daily with 5% D-sorbitol treatment for 15 min at 37 °C and/or *N*-acetylglucosamine included in the culture medium for duration of the sampling.

All cultures were maintained with daily medium changes and monitored with Giemsa-stained thin smear microscopy and parasite stage distribution determined by counting ≥100 parasites per day. Parasite samples (30 ml of 2–3% gametocytemia, 4–6% hematocrit) were harvested daily for microarray analysis on days − 2 to 13 following gametocyte induction. The samples harvested on days − 2 to 7 were isolated from uninfected erythrocytes via 0.01% w/v saponin treatment for 3 min at 22 °C while samples from day 8 to 13 were enriched for late stage gametocytes via density centrifugation using Nycoprep 1.077 cushions (Axis-Shield). Late stage gametocyte samples were centrifuged for 20 min at 800×*g* and the gametocyte containing bands collected [74]. All parasite samples were washed with phosphate-buffered saline before storage at − 80 °C until RNA was isolated, comprising a single full biological replicate of the time course. The time course allows detection of experimental dynamic changes and inform sequential analyses to indicate validity of the data.

### RNA isolation, cDNA synthesis and microarray hybridization and scanning

Total RNA was isolated from each parasite pellet with a combination of TRIzol (Sigma Aldrich, USA) treatment and using a Qiagen RNeasy kit (Qiagen, Germany) as per manufacturer’s instructions. The quantity, purity and integrity of the RNA were evaluated by agarose gel electrophoresis and on a ND-2000 spectrophotometer (Thermo Scientific, USA). For each RNA sample, 3–12 μg total RNA was used to reverse transcribe and dye couple cDNA as described previously [44]. The reference cDNA pool was constructed from a mixture of all the gametocyte samples used in the experiment in a 1:4 ratio with cDNA from a 6-hourly time course of asexual *P. falciparum* 3D7 parasites. For microarray hybridization, equal amounts of cDNA between 150 and 500 ng of Cy5 labeled sample and Cy3 labeled reference pool were prepared for hybridization as described previously [44]. Arrays were scanned on an Agilent G2600D Microarray Scanner (Agilent Technologies, USA) with 5 μm resolution at wavelengths of 532 nm (Cy3) and 633 nm (Cy5). Linear lowess normalized signal intensities were extracted using the Agilent Feature Extractor Software version 11.5.1.1 using the GE2_1100_Jul11_no_spikein protocol and data was uploaded onto the Princeton University Microarray Database (https://puma.princeton.edu/).

### Data analysis

Signal intensities loaded on the Princeton University Microarray Database were filtered to remove background and unsatisfactory spots were flagged for removal using spot filters *P* < 0.01 and log_2_ (Cy5/Cy3) expression values were used for further analysis. Euclidean distance clustered heatmaps were generated using TIGR MeV software version 4.9.0 (http://www.tm4.org/mev.html). The R statistical package (version 3.3.2) was used to calculate Pearson correlation coefficients and these were visualized using the Corrplot package. Data were divided into 10 clusters using K-means analysis following a within sum of squares test to determine the optimal number of clusters.

For functional analysis of genes, gene ontology enrichments were obtained for biological processes with *P <* 0.05 using curated evidence using PlasmoDB Release v 33 (http://www.plasmodb.org/) and supplemented with annotation from MPMP [68] and Interpro (https://www.ebi.ac.uk/interpro/). Additional datasets for translationally repressed genes [33, 35], transcripts involved in commitment [18, 20, 25] and gametocyte transcriptomes and proteomes [35, 39–41] were probed for significant association with clusters of expression using a two-tailed Fisher’s exact test to calculate significant association between the datasets. For comparison between transcript abundance and histone post-translation modifications, supplementary information was sourced from published histone PTM mass spectrometry data [57] done on multiple stages of parasite development on the same strain of parasites used in this study and specific localization of these PTMs were sourced from ChIP-seq or ChIP-chip experiments from the Gene Expression Omnibus (GEO) datasets for H3K56ac, H4K5/8/12 ac [75] as well as Salcedo-Amaya et al. for H3K9me3 [17], Jiang et al. 2014 for H3K36me3 [16] and Flueck et al. 2009 and Fraschka et al. for HP1 occupancy in *P. falciparum* parasites [47, 48]. The genes associated with the specific histone marks in each of the publications were then probed for association with specific clusters of expression using two-tailed Fisher’s exact tests and increased presence of the post-translational modification in gametocytes [57]. To determine the involvement of ApiAP2 transcription factors in gametocyte development, the Gene Regulation Network Inference Using Time Series (GRENITS) package in R was applied (probability threshold > 0.7) using the total transcriptome as possible regulated genes [71]. The package uses Dynamic Bayesian Networks and Gibbs Variable Selection to construct a linear interaction model between gene expression profiles of putative “regulators” and “regulatees” over time-correlated data. Following the identification of 5 ApiAP2 transcription factors (*ap2-g* was not included in further predictive analysis) with putative regulatory activity, these transcription factors were re-probed as regulators, using genes containing the transcription factor’s binding site as possible regulated genes if the binding site had been determined [13]. The number of links per model, per threshold was evaluated to determine the set probability threshold for the regulated genes of each transcription factor. The online FIRE algorithm [54] was used to identify enriched regulatory motifs in genes of interest in specific clusters of genes.

### qPCR validation of gametocyte time course microarray

RNA samples were obtained from stage II (early-stage gametocytes) and stage V (late-stage gametocytes). The seryl-tRNA synthetase (*pf3d7_0717700*) (IDT, USA) (forward primer sequence 5’TTCGGCAGATTCTTCCATAA-3′, and reverse primer sequence 5′-AAGTAGGAGGTCATCGTGGTT-3′) was used as reference gene. The primers used for each of the genes were: *pf3d7_0406200* (*pfs16),* forward: 5′- TGCTTATATTCTTCGCTTTTGC-3′, reverse: 5′- TAGTCCACCTTGATTAGGTCCA-3′, *pf3d7_0422300* (*α tubulin II),* forward: 5′- ATCAATTATCAGCCCCCTAC-3′, reverse: 5′- GCCCTTTTCGCATACATC-3′, *pf3d7_0816800* (*dmc1*)*,* forward: 5′-GGAATTGTCTGAGAGGCAAC-3′, reverse: 5′- ACTGGTTTCATTGGGTTAGC-3′. Real time quantitative PCR (qPCR) was conducted using the 2X PowerUP SYBRGreen Master Mix (Thermo Fisher Scientific, USA) kit in white 384 well plates and analyzed using the QuantStudio 12 K Flex system (Life Technologies, USA). The reaction was run according to the manufacturer’s instructions from 2 ng cDNA for 40 cycles. For relative quantification the ^2−^ΔCt method was used to calculate of the difference in expression of the gene of interest compared to the reference gene (75). Data were subsequently expressed as log_2_FC (EG/LG) (Additional File 1).

## Additional Files

**Additional File 1: Table S1** Total microarray data with GO enrichment pertaining to Fig. 1 & 2

**Additional File 2.** Correlation of microarray time points and gametocyte markers pertaining to Fig. 1

**Additional File 3.** Cross-dataset comparison and functional enrichment pertaining to Figs. 2-5

**Additional file 4: Fig. S1**. qPCR validation of select gametocyte genes. **Fig. S2**. Transcript abundance of ApiAP2 transcription factors during *P. falciparum* gametocyte development. **Fig. S3**. Transcript abundance of *ap2-g* and downstream genes (identified in Josling et al. 2019)

## Data Availability

The dataset supporting the conclusions of this article is available in the Gene Expression Omnibus (GEO) repository, with accession number GSE104889 (www.ncbi.nlm.nih.gov/geo/).

## Abbreviations

ApiAP2: Apicomplexan APetala 2
ARID: AT-rich Interaction Domain
CITH: Trailer Hitch Homolog
DOZI: ATP-dependent RNA helicase DDX6
FIRE: Finding Informative Regulatory Elements
GDV1: Gametocyte Development Protein 1
GEO: Gene Expression Omnibus
GO: Gene Ontology
GRENITS: Gene Regulatory Network Inference Using Time Series
HC: Hematocrit
HP1: heterochromatin protein 1
hpi: Hours Post Invasion
IDC: Intraerythrocytic developmental cycle
LysoPC: Lysophosphatidylcholine
NAG: N-Acetyl Glucosamine
P: Parasitemia
PTM: Histone Post-Translational Modification
PUF2: Pumilio Family protein 2
SERA: Serine Repeat Antigens
UTR: Untranslated Region
