# Supplementary material for: Hierarchical transcriptional control regulates Plasmodium falciparum sexual differentiation
Source: BMC Genomics. 2019 Dec 3;20:920. doi: 10.1186/s12864-019-6322-9 (PMC6889441; doi:10.1186/s12864-019-6322-9)
Supplement: Supplementary file 4 — Additional file 4: Fig. S1. qPCR validation of select gametocyte genes. Fig. S2. Transcript abundance of ApiAP2 transcription factors during P. falciparum gametocyte development. Fig. S3. Transcript abundance of ap2-g and downstream genes (identified in Josling et al. 2019) [file 12864_2019_6322_MOESM4_ESM.pdf]

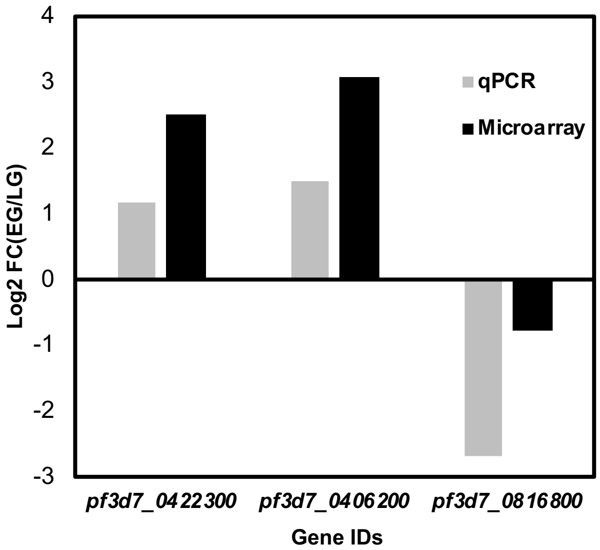

Additional Figure 1. qPCR validation of select gametocyte genes

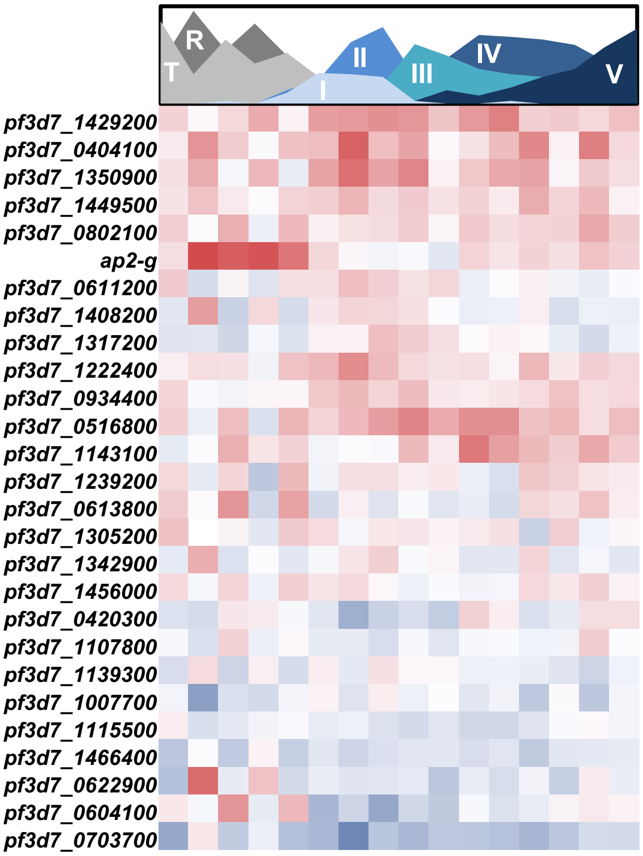

Additional Figure 2. Transcript abundance of ApiAP2 transcription factors during *P. falciparum* gametocyte development

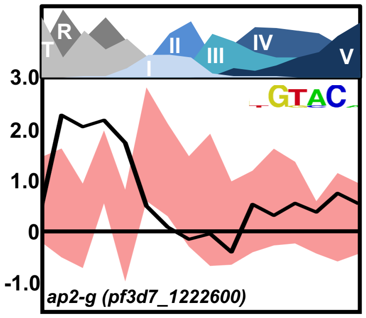

Additional Figure 3. Transcript abundance of *ap2-g* (line) and downstream genes (ribbon, identified in Josling *et al.* 2019)
